# Subsidence of a metaphyseal-anchored press-fit stem after 4-year follow-up: an EBRA-FCA analysis

**DOI:** 10.1007/s00402-021-04068-8

**Published:** 2021-07-21

**Authors:** Dietmar Dammerer, Philipp Blum, David Putzer, Dietmar Krappinger, Michael C. Liebensteiner, Michael Nogler, Martin Thaler

**Affiliations:** 1 Department of Orthopaedics and Traumatology, Medical University of Innsbruck, Anichstrasse 35, 6020 Innsbruck, Austria; 2 Department of Experimental Orthopaedics, Medical University of Innsbruck, Sonnenburgstr. 16, 6020 Innsbruck, Austria

**Keywords:** Stem subsidence, Total hip arthroplasty, Cementless, Einzel-Bild-Röntgen-Analyse (EBRA)

## Abstract

**Purpose:**

Uncemented stem migration analysis by EBRA-FCA (Einzel-Bild-Roentgen Analyse, Femoral Component Analyse) has been seen to be a good predictive indicator for early implant failure. In this study, we investigated the migration behavior of a cementless metaphyseal-anchored press-fit stem after 4-year follow-up.

**Methods:**

Applying a retrospective study design, we reviewed all consecutive patients who between 2012 and 2017 received a cementless Accolade II press-fit stem at our Department. We reviewed medical histories and performed radiological measurements using EBRA-FCA software. EBRA-FCA measurements and statistical investigations were performed by two independent investigators.

**Results:**

A total of 102 stems in 91 patients (female 60; male 31) fulfilled our inclusion criteria. Mean age at surgery was 66.2 (range 24.3–92.6) years. EBRA migration analysis showed a mean subsidence of 1.4 mm (range 0.0–12.0) at final follow-up. The angle between stem and femur axis was 0.5° (range 0.0°–2.8°) after 48 months. No correlations between gender or Dorr types and subsidence were found (*p* > 0.05). A body mass index > 30 kg/m^2^ showed a significant increase in stem subsidence within the first 6 (*p* = 0.0258) and 12 months (*p* = 0.0466) postoperative.

**Conclusions:**

Migration pattern of the metaphyseal-anchored stem and a low subsidence rate at final follow-up may predict a good long-term clinical result.

**Trial registration:**

Number: 20181024-1875.

## Introduction

Cemented as well as cementless femoral components in total hip arthroplasty (THA) yielded excellent long-term survival rates over 95% after 10 years [1]. Due to the literature, already 6 months after implantation of a primary cementless THA, aseptic loosening is described as the most common cause of failure in THA [2–4]. Previously published studies reported distal migration of the femoral stem, called subsidence, which has shown to be a good predictive factor for early aseptic component loosening [5–8]. According to Krismer et al., distal migration of the stem of more than 1.5 mm (mm) detected with Einzel-Bild-Roentgen Analyse–Femoral Component Analysis (EBRA-FCA) within the first 2 years is a well-established risk factor for early implant failure [9]. However, comparability is limited due to the inclusion of cemented and cementless stems by Krismer et al. [9].

EBRA-FCA is a computer-assisted method for measuring the distal migration of femoral stems using standard anterior–posterior (ap) pelvic radiographs without requiring additional means at exposure (e.g. ball markers). It has proven accuracy and a sensitivity of more than 1 mm in detecting migration as compared to RSA (roentgen stereophotogrammetric analysis) [10, 11].

The stem used in this study is the Accolade II by Stryker^®^ (Stryker, Kalamazoo, MI, USA). It is designed for cementless, press-fit application and has a morphologic wedge with a size-specific medial curvature and a hydroxyapatite-coated proximal region [12]. As example given to the numerous arthroplasty registers around the world, in the Australian Orthopaedic Association National Joint Replacement Registry, 1961 Accolade II stems were implanted in 2018, which makes it the fifth most commonly used cementless stem in primary THA [13].

In the present study, we investigated the clinical results and the migration behavior of the cementless Accolade II stem using EBRA-FCA with a mid-term follow-up of up to 48 months. Furthermore, we evaluated the possible influence of gender, BMI and structural bone quality of the proximal femur on stem subsidence.

## Materials and methods

The study was approved by the local ethics committee (Medical University of Innsbruck, Austria, Europe). We applied a retrospective study design and reviewed all consecutive patients who received an Accolade II stem at our department between 2012 and 2017. During this time, a total of 219 Accolade II stems were implanted as part of a primary THA.

We investigated the medical histories for sociodemographic data, surgical approach, body mass index, cut-to-suture time and the preoperative diagnosis for THA indication. Furthermore, range of motion was recorded preoperatively and up to 1 year after surgery by surgeons at our department using a goniometer during clinical examination.

**Fig. 1:**
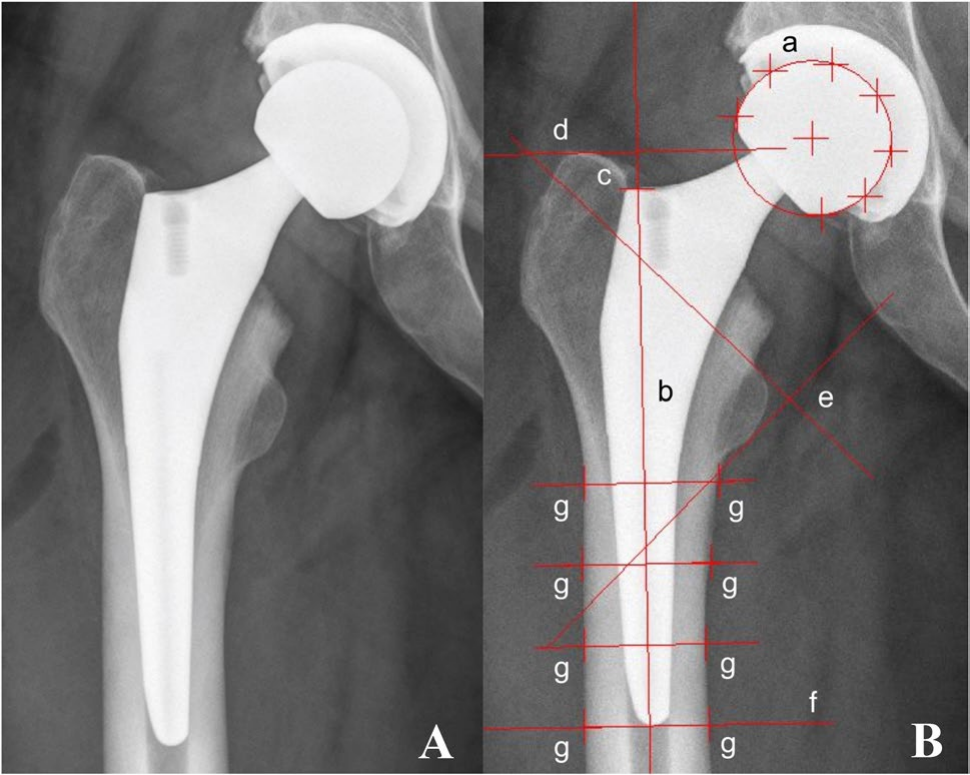
Anterior to posterior X-rays showing an Accolade II stem (**A**) and with EBRA-FCA references (**B**) a head points b stem axis c stem shoulder d major trochanter line e minor trochanter lines f tip-of-stem line g points at femoral bone contour

Axial stem migration, prosthetic stability as well as tilting of the stem were assessed retrospectively with EBRA-FCA from plain X-rays [9, 10]. A total of 19 reference points are defined on the femoral head (*n* = 7), the stem (*n* = 2), the femoral cortex (*n* = 8), and 1 each at the major and minor trochanter [10]. The EBRA-FCA software excludes radiographs with a comparability algorithm, which identifies significant positioning artifacts by comparing specific bone and prosthetic landmarks. Figure 1 shows the X-ray of an Accolade II stem including EBRA-FCA references.

In our department, we routinely follow patients with radiographs before discharge, 6 weeks after surgery, 12 months postoperative and then in a 1–2 year interval. We perform additional radiographs if the patient has any complaints with the THA. All radiographs were taken at our Department of Radiology with the same technique [anterior–posterior (AP) radiographs; patient standing in upright position and full weight bearing]. For our EBRA investigation, a minimum of four radiographs per patient and a minimum radiological follow-up of up to 6 months was required for this analysis. Migration analysis was done with EBRA by one independent investigator, who was not involved in the surgeries or postoperative treatment of patients.

Our postoperative protocol allows immediate full weight bearing depending on pain after cementless primary THA from day one after surgery.

### Statistics

Mean, median, range, and standard deviation were calculated for the various measurement parameters. For the analysis, Access and Excel (Microsoft Office Professional Plus 2010, Redmond, WA, USA) as well as Graph Pad Prism (Version 8.0, GraphPad Software, Inc., La Jolla, CA, USA) were used. All data were tested for normality using the Kolmogorov–Smirnov test. For comparison of the EBRA-FCA measurements at different time steps as well as for comparison of subsidence according to the Dorr classification the Kruskal–Wallis test was used. The EBRA measurements were compared by BMI and gender classification using the Mann–Whitney *U* test. When comparing the range of motion pre- and postoperatively, the Mann–Whitney *U* test was used for comparison. A *p* value of 0.05 was considered statistically significant.

## Results

A total of 102 stems in 91 patients (female 60; male 31) fulfilled our inclusion criteria. In 11 patients, the Accolade II stem was implanted bilaterally. Mean patient age at surgery was 66.2 (range 24.3–92.6) years and mean body mass index was 27.2 (range 16.2–40.1) kg/m^2^. Mean follow-up was 48 (range 10–65) months. The preoperative diagnosis was osteoarthritis in 94 (92.2%) hips, avascular necrosis of the femoral head in 7 (6.8%) hips and a pathologic fracture in 1 (1.0%) hip. The mean cut-to-suture time was 63 (range 29–161) min. The investigated stem was combined with cementless press-fit cups. All surgeries were performed in a supine position using the direct anterior approach [14]. The most used head size was 32 mm (62.7%). More details are shown in Tables 1 and 2.

**Table 1:** Patient demographics for the study group

Number of patients	
Female	60
Male	31
Total	91
Mean age (years)	66.2 (24.3–92.6)
BMI (kg/m^2^)	27.2 (16.2–40.1)
Cut-to-suture time (min)	63 (29–161)
Surgical approach	
Direct anterior approach	102
Surgical position	
Supine	102
Preoperative diagnosis	
Osteoarthritis	94
Avascular necrosis of the femoral head	7
Pathologic fracture	1
Dorr classification	
Type A	14
Type B	83
Type C	5

Range is given in brackets

**Table 2:** Details of implanted components

Stem product	
Stryker Accolade II	102 [100.0]
CCD angle	
127°	80 [78.4]
132°	22 [21.6]
Head size (mm)	
22	1 [1.0]
26	1 [1.0]
28	30 [29.4]
32	64 [62.7]
36	6 [5.9]
Cup product	
Stryker Trident PSL	101 [99.0]
DePuy Bantam	1 [1.0]

Percentages are given in brackets

EBRA-FCA analysis at 48 months follow-up was calculated for 50 out of 102 stems with an EBRA-FCA-given comparability limit of 3.0 mm (95% confidence interval). A total of 637 x rays were analyzed, whereby 44 radiographs (6.9%) were rejected by the EBRA-FCA software. On average, 6.2 (range 4–20) x rays per implant were analyzed. None of our patients had to be excluded from EBRA-FCA migration analysis. A complete set of radiographs at every single time step (e.g. 6 months, 12 months, etc.) was not available for each stem in our study. Therefore, total subsidence could not be calculated in all cases. This gives a different number of cases in the corresponding migration behavior analysis over time.

**Fig. 2:**
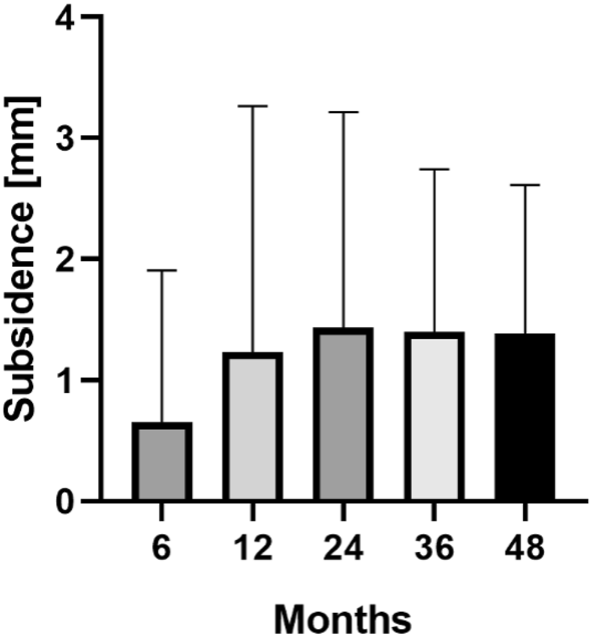
Mean and standard deviation (bars) of total stem subsidence for the clinical follow-up of 48 months

The EBRA-FCA analysis showed a mean migration of 0.7 mm (range 0.0–8.5) at 6 months, 1.2 mm (range 0.0–12.0) at 12 months, 1.4 mm (range 0.0–11.0) at 24 months, 1.4 mm (range 0.1–6.6) at 36 months and 1.4 mm (range 0.1–5.5) at 48 months after surgery. Thus, the main axial subsidence occurred particularly in the first postoperative year (Table 3; Fig. 2). The calculated mean axial implant migration was 0.11 mm in the first 6 months, 0.10 mm between 6 and 12 months, 0.02 mm between 12 and 24 months and 0.00 mm thereafter.

**Table 3:** Mean total subsidence in millimeters (mm) over time

	6 months (*n* = 100)	12 months (*n* = 39)	24 months (*n* = 64)	36 months (*n* = 66)	48 months (*n* = 50)
Subsidence of the Accolade II stem in mm (range)	0.7 (0.0–8.5)	1.2 (0.0–12.0)	1.4 (0.0–11.0)	1.4 (0.1–6.6)	1.4 (0.1–5.5)

Range is given in brackets

**Fig. 3:**
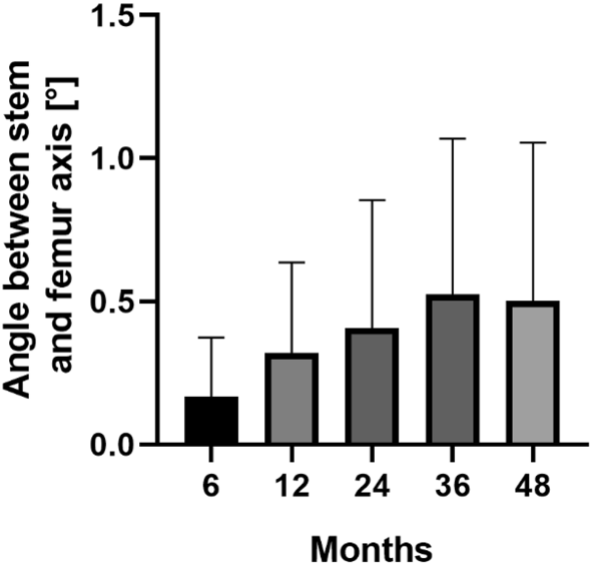
Mean and standard deviation (bars) of the angle between stem and femur axis for the clinical follow-up of 48 months

In addition, the angle between stem and femur axis was 0.2° (range 0.0°–0.9°) after 6 months, 0.3° (range 0.0°–1.5°) after 12 months, 0.4° (range 0.0°–1.7°) after 24 months, 0.5° (range 0.0°–2.6°) after 36 months and 0.5° (range 0.0°–2.8°) after 48 months (Fig. 3).

A statistically significant smaller angle was found at 6 months in comparison to 12 months (*p* = 0.0191), 24 months (*p* = 0.0011), 36 months (*p* < 0.0001) and 48 months (*p* < 0.0001). Also, statistically significant less initial subsidence was found at 6 months in comparison to 12 months (*p* = 0.0235), 24 months (*p* < 0.0001), 36 months (*p* < 0.0001) and 48 months (*p* < 0.0001).

**Fig. 4:**
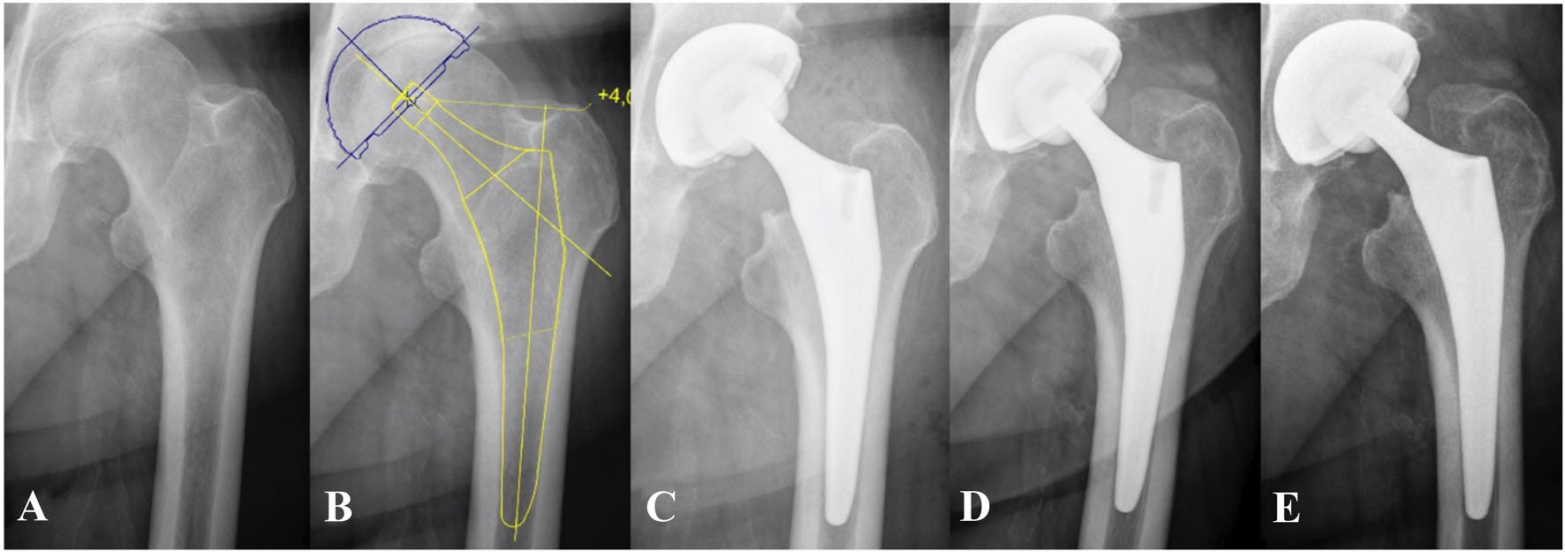
The X-ray series presents the procedure of the patient with the largest detected subsidence **A** preoperative situation showing osteoarthritis of the hip **B** preoperative prosthetic planning **C** immediate postoperative X-ray **D** subsidence of 5.3 mm was detected by EBRA-FCA 6 weeks after surgery **E** subsidence of 12 mm was detected by EBRA-FCA 12 months after surgery

Of 103 stems 64 had sufficient EBRA-FCA follow-up to assess migration behavior after 2 years. Working from the critical threshold values, 18 (28.2%) of 65 stems showed an axial subsidence of more than 1.5 mm. An axial subsidence of more than 2.7 mm was detected in nine (14.1%) of 64 cases 2 years postoperatively. Percentages of migrated stems are given in Table 4. The most severe stem subsidence (12.0 mm) was observed in a patient within the first 12 months. Postoperative X-rays of this patient were re-evaluated and undersizing of the stem was identified as probable cause for subsidence. However, no postoperative complications were observed in this case, e.g. stem revision, limping or pain. The procedure for this patient is shown in Fig. 4. Additionally, another implant showed a subsidence of 11 mm after 24 months. In this case, undersizing was ruled out and no postoperative complications (e.g. stem revision, etc.) were documented.

**Table 4:** Total subsidence in millimeters (mm) over time

Total subsidence (mm)	6 months (*n* = 100)	12 months (*n* = 39)	24 months (*n* = 64)	36 months (*n* = 66)	48 months (*n* = 50)
≤ 1.5	89 (89.0)	30 (76.9)	46 (71.8)	44 (66.7)	32 (64.0)
> 1.5	6 (6.0)	5 (12.8)	9 (14.1)	13 (19.7)	12 (24.0)
> 2.7	5 (5.0)	4 (10.3)	9 (14.1)	9 (13.6)	6 (12.0)

Percentages are given in brackets

According to the Dorr classification, we divided our patient cohort into three groups to measure its effect on subsidence [15]: patients with femoral bone classification Type A, Type B and Type C. There was no statistically significant difference in the total migration between the three sub-cohorts (*p* > 0.05). Nevertheless, the stem subsidence in Type C femurs was higher than the overall mean subsidence at last measurement in 3/5 (60%) stems. A statistically larger angle was found at 6 months for Type A in comparison to Type C femuras (*p* = 0.0265). Additionally, the patients were divided into groups according to their BMI: normal (BMI ≤ 25 kg/m^2^), overweight (BMI 25.1–29.9 kg/m^2^) and obese (BMI ≥ 30 kg/m^2^). A statistically significant greater subsidence of the stem was found for obese patients within 6 months (*p* = 0.0258) and within 12 months (*p* = 0.0466) of surgery in comparison to normal and overweight patients. No statistically significant difference was found between obese and normal to overweight patients when considering the variation in the stem angle. Furthermore, EBRA measurements for stem angle variations and subsidence between female and male patients showed no statistically significant difference (*p* > 0.05).

Pre- and postoperative comparison of the range of motion showed a mean improvement in flexion of 10° (range − 45° to 40°, *p* < 0.0001), internal rotation 13° (range − 20° to 40°, *p* < 0.0001), external rotation 8° (range − 20° to 40°, *p* < 0.0001), abduction 13° (range − 30° to 45°, *p* < 0.0001) and adduction 11° (range − 20° to 30°, *p* < 0.0001). Preoperatively, flexion ≥ 90° was possible in 69.9% of the investigated hip joints; this figure increased to 93.2% postoperatively.

In addition, postoperative complications were documented. In four cases (3.9%), a fracture occurred after implantation. In another five cases (4.9%), a revision procedure had to be performed. Of these, four cases showed migration > 1.5 mm and thereof three cases > 2.7 mm within the first 2 years. Two cases of revision were carried out in Dorr Type C femurs. No infection was observed overall.

## Discussion

As THA is one of the most successful medical procedures, various stem designs are available. Short stems have been clinically established for years with significant differences between femoral fixation concepts [16]. The present study analyzed the migration behavior of the cementless methaphyseal-anchored Accolade II stem. To the best of our knowledge, this is the first study to investigate the mean subsidence of the Accolade II stem using EBRA-FCA. After a follow-up of 48 months, we can report a low mean subsidence of 1.4 mm and a small axial deviation of 0.5° between stem and femur axis. In addition, more than 85% of the stems showed a subsidence of less than 2.7 mm and, although more than 70%, less than 1.5 mm after 24 months. Therefore, our results are well in line with previously published EBRA-FCA studies, and thus good long-term implant survival of the investigated femoral stem may be expected.

Primary stability is a prerequisite for the bony ingrowth of the implant in THA. Working from this assumption, increased subsidence of the femoral component within the first 2 years after surgery was described as a risk factor for later aseptic loosening [9]. With a specificity of 100% and a sensitivity of 78% for detection of migration of more than 1 mm, as compared with roentgen stereophotogrammetric analysis (RSA), EBRA-FCA is suitable for identifying and measuring the subsidence of femoral components in THA [10]. While RSA is considered to be the gold standard for migration measurement, EBRA-FCA offers the advantage of being a non-invasive method that can be used in our retrospective study design.

Different thresholds for prediction of aseptic loosening have been described in the given literature [5, 9]. In our study, 28.2% of the stems showed a migration of more than 1.5 mm and 14.1% of more than 2.7 mm 2 years postoperatively. Similar results were recently published by Kutzner et al. for the calcar-guided Optimys stem (Mathys Ltd Bettlach, Bettlach, Switzerland), stating that 38.2% and 15.2%, respectively, showed a subsidence of more than 1.5 mm and 2.7 mm after 2 years [17]. Tian et al. also investigated the Accolade II stem, with only one of 1017 (0.1%) implants subsiding more than 1.5 mm after 2 years [18]. While in previous mentioned studies, EBRA-FCA was used to determine subsidence, the measurement method used by Tian et al. cannot be exactly followed from their study protocol. The different measurement methods might be a reason for the large differences in detected subsidence after 2 years.

Migration analysis of various metaphyseal-anchored femoral short stems has already been part of several short- to mid-term EBRA studies (15–20) [17, 19–23]. Kutzner et al. showed a mean subsidence of 1.39 mm after 2 years and 1.5 mm after 5 years for the Optimys stem [17]. When investigating the same stem, Schaer et al. reported a slightly larger mean axial migration of 1.71 mm and 2.04 mm after 2 and 5 years, respectively [22]. While Schmidutz et al. found a mean subsidence of 0.7 mm after 2.7 years for the Metha stem (B. Braun Aesculap, Tuttlingen, Germany), a mean subsidence of 1.01 mm 2 and 3 years after surgery was reported by Jahnke et al. for the same stem [20, 23]. In a study by Freitag et al., the Fitmore stem (Zimmer Biomet, Warsaw, IN, USA) showed 1.1 mm of axial migration after 2 and 5 years [21]. As compared to these results, mean subsidence in our study was 1.4 mm after 2 and 4 years. Nearly all the above-mentioned implants showed initial subsidence with secondary stabilization after 2 years. This phenomenon, already described by Krismer et al. in 1999, is not uncommon and can lead to long-lasting survival of the implant [9]. Main subsidence of the Accolade II stem was observed during the first postoperative year. Mean monthly migration reduced from 0.11 mm during the first 6 months after surgery to less than 0.02 mm after 24 months. As a result, a stable implant situation between 1 and 2 years postoperatively can be assumed.

Several factors potentially affecting the subsidence of femoral components have already been investigated. The influence of patient demographics like weight, BMI and gender on stem subsidence yielded different results. Stihsen et al. observed a significant impact of body weight over 75 kg on subsidence of the Vision 2000 stem (Depuy, Warsaw, IN, USA) [24]. These findings were confirmed by Kutzner et al. for the Optimys stem [25]. In contrast to body weight, a BMI > 30 kg/m^2^ had no influence on subsidence in previous studies [24, 25]. However, in a study by Freitag et al., the Fitmore stem showed a tendency to greater subsidence in patients with a BMI > 30 kg/m^2^ without finding significant differences [26]. In our study, we demonstrate a statistically significant greater subsidence of the Accolade II stem in obese patients within 6 months (*p* = 0.0258) and 12 months (*p* = 0.0466) of follow-up in comparison to normal and overweight patients. While some studies showed male gender to be at risk for greater subsidence [17, 24], we cannot report a statistically significant difference between female and male patients.

In addition to patient demographics, press-fit of the stem is considered to be one of the key factors [27]. A connection between femoral configuration and axial migration was detected by Jahnke et al. for the Metha short stem [28]. In our study, the preoperative Dorr types of femoral configuration showed no significant influence on subsidence of the Accolade II stem, whereby stem subsidence in Type C femurs was higher than mean subsidence in 60%. The subsidence of the Optimys stem was also not influenced by the Dorr types, but Schaer et al. found a correlation between Optimys stem size and subsidence, with stem size ≥ 6 showing a larger subsidence than stem size < 6 at 5-year follow-up [22]. However, this phenomenon was observed only in women. Schaer et al. concluded that the surgeon may not have chosen an even larger implant out of fear of an intraoperative periprosthetic fracture, as women tend to require smaller implants than men [22].

This study has several limitations, including the absence of a control group and retrospective methodology. As a result, some of the treated patients had to be excluded from the cohort, possibly making the study more prone to selection bias. Nevertheless, our number of cases is larger than in some of the cited studies [20–23]. Therefore, valid results can be assumed. Furthermore, the number of radiographs and duration of follow-up varied for each hip. This may have influenced the migration results due to the smoothing function in the software and made it difficult to follow the exact outcome of each individual implant. Due to the retrospective character of this study, we could not rule out all factors influencing stem subsidence. As already known, stem subsidence is multifactorial influenced by e.g. BMI, undersizing the stem etc.; all these factors may be a contributor for early migration. Especially worth mentioning is undersizing, a well-known obstacle and problem by using a metaphyseal-anchored press-fit stem in THA. This problem could not be ruled out in all cases because of the retrospective study character. To undersize a femoral stem is and will be a common problem in THA, nevertheless in some cases, the stem will stay in place over years and in some other cases, the stem will migrate very early. We, therefore, advice the surgeon to be aware of this problem and should have a more frequently follow-up of these patients. In addition, the number of Dorr C type femurs may have been too small to detect significant differences and a power analysis was not performed.

In summary, the EBRA-FCA analysis for the cementless Accolade II short stem showed a small mean subsidence and low mean tilting of the stem axis with good clinical function up to 4 years after surgery. After an initial subsidence in the first postoperative year, the mean monthly migration rate reduced and a stable implant position may be subsequently predicted. While gender and Dorr classification had no influence on subsidence, a statistically significant greater subsidence of the stem was found for obese patients within 6 months and 12 months. Further observations are necessary to confirm the expected good long-term results.

## Data Availability

Data will be sent if necessary.
